# How providing public COVID-19 mitigation instructions in a foreign language can increase people’s sense of control

**DOI:** 10.1371/journal.pone.0277366

**Published:** 2022-11-23

**Authors:** Katharina Saile, Rafael Munz, Verena Hüttl-Maack

**Affiliations:** Chair of Marketing & Consumer Behavior, Institute of Marketing & Management, University of Hohenheim, Stuttgart, Germany; Nagoya University, JAPAN

## Abstract

Processing information in a learned foreign language can alter one’s judgment or cognitive evaluation of stimuli. Documented consequences include a reduction in perceived negativity and perceived severity of crime or diseases. The global COVID-19 pandemic has offered a unique opportunity to investigate this phenomenon in a real-life public health communication context. The aim of this study is to investigate how foreign language processing influences people’s reaction towards freedom-restrictive messages. In our experimental study (*N* = 605), we presented participants with pandemic mitigation instructions in their native language versus a learned foreign language and assessed their perceived sense of control, cognitive evaluation of the instructions, and the intention to adhere to them. The results indicated that the use of a foreign language influenced people’s perceived sense of control in a way that might intuitively be surprising: foreign language enhanced sense of control. This positively influenced the cognitive evaluation of the instructions’ effectiveness and the intention to comply with them. The present research demonstrates that foreign language processing influences individuals’ responses to specific, real-life instructions. Our results provide important contributions to the literature on foreign language effects and public communication and enable practitioners to more accurately predict recipient responses to global crisis communications.

## Introduction

When navigating the coronavirus pandemic (COVID-19) after its outbreak in 2019, governments, institutions, brands, and service providers have frequently issued new pandemic mitigation instructions. These public actors have aimed to convince citizens to adhere to the new rules in order to ensure public safety. However, this was not always a given: press reports have documented examples from many countries, indicating that countless people have criticized and defied public health instructions [e.g., 1, 2]. Scholarly research shows that these public health messages are perceived and reacted to in different ways by individuals. It has been posited that people want to receive clear instructions on how to act to protect themselves and others when faced with threats [3]. However, recent studies have also shown that such freedom-threatening messages can elicit negative reactions in recipients, leading, for example, to a lower intention to behave in a socially responsible manner and greater psychological reactance [4]. Similarly, public health messages perceived as threatening can elicit highly negative emotional reactions [5].

When looking beyond messages, the pandemic, with all its consequences, has negatively affected the well-being of many people. Poorer sleep quality [6] and increased suicidal thoughts during lockdowns [7] are clear indicators of this development. Crisis hotline calls have spiked during the COVID-19 pandemic, even more so, when new mitigation instructions have been communicated [8]. The COVID-19 pandemic and its accompanying restrictions on peoples’ lives is the epitome of the type of situation in which many people experience feelings of a lack of personal control [9]. One key reason is that the measures taken to mitigate the pandemic are very intrusive regarding people’s personal lives. For instance, the citizens of many countries have experienced the need to reduce social contacts and adhere to lockdown regulations. In line with this, it has been proposed that especially, but not only, adolescents have experienced a strong loss of autonomy and freedom due to restrictions during the pandemic [10]. If individuals cannot behave as they wish, their sense of control over their environment and behavior is threatened [11]. However, the maintenance and pursuit of personal control is one of humankind’s central motivations [12]. This sense of control is sometimes framed as a feeling of dominance, which relates to feeling influential and in control over one’s own environment, in contrast to experiencing a total lack of control [13, 14]. Due to the importance of people experiencing control over their own lives for their well-being and motivation for action [e.g., 15, 16], studying how a sense of control can be influenced seems essential.

Our study aimed to use knowledge of how foreign languages affect human perception and judgment to better understand people’s reactions to restrictive public health messages and instructions that affect people’s sense of control. Based on findings from foreign language research, we assume that using a foreign language can reduce feelings of a lack of control. Empirical evidence shows that processing stimuli in a foreign language can lead to a reduction in perceived negativity, such as less severe judgments of medical conditions [17] or crimes [18]. Most recently, one study even investigated the negativity-reducing influence of foreign language on COVID-19 vaccine attitudes [19]. The results of this study indicate that by increasing trust, foreign language reduces hesitancy against corona virus vaccination. Against this background, the research question of this study was whether people react differently to public health messages in a foreign language compared to their native language. More specifically, we investigated whether processing restrictive pandemic mitigation instructions in a foreign language (vs. a native language) could lead to a higher sense of control. In addition, we examined whether this enhanced sense of control can improve the cognitive evaluation of the effectiveness of the instructions and the motivation to comply with them.

Due to increased globalization and migration, this issue is of utmost relevance. Through the pandemic as a global crisis, individuals have been exposed to COVID-19-related content in English as a world language, for example, through social media. Importantly, this includes people from all over the world for whom English is not their native language. Additionally, with increasing migration, many people are confronted with public communication in a language that is foreign to them.

In the following section, we provide a theoretical background grounded in foreign language research and close with our predictions regarding expected effects. We then present an empirical study in which these predictions were tested and discuss our results. Finally, we outline how this research contributes to the extant literature, how practitioners can make use of our findings, and the existing limitations of our study.

### Conceptual background

#### Foreign language processing

Research on foreign language processing has been conducted in a variety of contexts. Until now, prior research has often investigated the influence of foreign versus native language on cognitive evaluations and decision-making. This research has mostly studied rather abstract and hypothetic scenarios, such as the trolley dilemma [20], fiction book excerpts [21], the Asian disease problem [22], and bad luck/superstition scenarios [23]. Only a few studies have applied a more realistic context to foreign language research [e.g., 17, 19, 24, 25]. However, to the best of our knowledge, no studies have investigated foreign language use in the context of public health communication.

Research results regarding the influence of foreign language on information processing, judgments, and behavior are diverse and inconsistent. So far, it is known that the use of a foreign language influences a variety of cognitive responses, such as decision-making or judgments, which lead to different outcomes compared to the use of a native language. However, the underlying mechanism of these differences has not yet been conclusively clarified in research.

Early research provided initial evidence that foreign language use might decrease the emotional intensity of a stimulus [e.g., 26–28]. It has been suggested that using a foreign language might promote increased analytical thinking, which could lead to the attenuation of emotions [e.g., 22, 29]. However, recent research has cast some doubt on the explanatory approaches of attenuated emotions or increased deliberation: For example, contrary to their hypothesis, Geipel et al. [20] found in their study that the attenuation of emotions did not drive the foreign language effect on moral judgment. As for the proposed increase in analytical thinking, Białek et al. [30] found evidence in the context of syllogistic reasoning that people who are reasoning in a foreign language compared to their native language even perform worse. The authors assume that these findings can be attributed to perceived difficulties identifying conflicts between competing intuitions in a foreign language. Additionally, Mækelæ and Pfuhl [31] did not find evidence that foreign language influences deliberate reasoning for an emotionally neutral task.

When specifically considering people’s reactions to pandemic mitigation instructions in a foreign language, we can draw assumptions from previous foreign language research in close-to-reality context studies. It turns out that foreign language use can change the evaluation of a stimulus or situation, in that it reduces the perceived negativity of the subject. Woumans et al. [18] found that crimes are evaluated as less severe in a foreign language than in a native language. The same holds for medical judgments: Hayakawa et al. [17] found that the use of a foreign language influences medical judgments. Medical conditions were perceived as less severe in a foreign language; in other words, they were judged as easier to cure, less physically painful, or less emotionally distressing. Geipel et al. [25] showed that the use of a foreign language increases consumers’ willingness to consume aversive products (e.g., recycled water, insect-based food) by reducing disgust. The perceived negativity of the products seems to be attenuated. Furthermore, the use of a foreign language has been shown to reduce the perceived negativity of the coronavirus vaccination in particular; in their study, Geipel et al. [19] found that the use of a foreign language reduces vaccination hesitancy by increasing trust.

In addition to a reduction of negativity, the use of a foreign language can also change the cognitive evaluation of the consequences of certain behaviors. This is shown by the results reported by Geipel et al. [32], who found that foreign language use shifts a person’s evaluation perspective from intention to outcome, which means that the ultimate consequences of a decision are considered more important than the measures taken to achieve those consequences. The authors presented participants with actions that had positive outcomes but dubious intentions in one study and actions with negative outcomes but positive intentions in the second study. Participants in the foreign language conditions reported more positive moral evaluations for the positive outcome scenario and more negative moral evaluations for the positive intentions scenario.

Results from Champoux-Larsson and Knežević Cvelbar [24] indicated first evidence in another field that people seem likelier to accept inconvenient consequences for themselves when presented in a foreign language. They investigated the influence of the use of a foreign language on a tourism-specific decision-making task. According to their results, the perceived negative personal consequences of a community-oriented yet personally inconvenient choice seem to be reduced. Participants in their study chose a utilitarian (environmentally friendly) option significantly more in a foreign language, while they chose a selfish and more convenient (less environmentally friendly) option more in their native language. Consumers may have found it easier to deal with the choice that was inconvenient for them when it was presented as such. The overall outcome of the inconvenient eco-friendly behavior may be more important than the selfish, convenient behavior itself.

### Predictions

Due to the confrontation with restrictive pandemic mitigation instructions, people seem to be experiencing multiple negative consequences [e.g., 6, 7]. In particular, the innate need to exert personal control, which is central for people’s well-being, is severely threatened and reduced by the pandemic [9]. The preceding considerations suggest that the use of a foreign language can, in fact, reduce the perceived negativity of a stimulus [e.g., 17–19, 25] and induce a cognitive focus towards the outcome of behaviors [32], both of which is likely to increase individuals’ sense of control. We apply this reasoning to the pandemic as a negatively valenced and threatening situation. Therefore, we propose that reading pandemic mitigation instructions in a foreign language will lead to a higher perceived sense of control compared to the native language.

When looking at the downstream consequences, we argue that a sense of control influences the cognitive evaluation and intention to comply with pandemic mitigation instructions. Sense of control is known to be a major driver of intention to behave in a certain way [e.g., 33]. Furthermore, a sense of control can increase a person’s capability to withstand distressing or aversive states [34], such as adhering to freedom-threatening pandemic mitigation instructions. Xu et al. [35] suggest that a high sense of control can enable people to manage the demands elicited by distressing situations. In their study, they showed that a high sense of control leads to higher motivation for charitable behavior. In other words, people seem to be willing to accept personal disadvantages in order to achieve a greater good. This can be transferred to the pandemic context, where people have to adhere to freedom-restricting mitigation instructions in order to achieve society’s overall well-being. This could be attributed to the fact that a high sense of control is often related to self-efficacy [e.g., 36]. Self-efficacy refers to the belief that one can perform certain actions that are required to achieve desirable outcomes [15, 37]. It seems likely that a higher sense of control motivates people to comply with pandemic instructions that are inconvenient and threatening yet good for society. We therefore hypothesize that sense of control positively mediates the relationship between language and cognitive appraisal in terms of the effectiveness of the instructions and the intention to comply with them.

## Methods

### Experimental design and stimuli

The study employed a between-subjects design with two conditions: native language (German) versus foreign language (English). All participants were presented with nine mitigation instructions related to the COVID-19 pandemic (see Table 1) in one of the two languages. Seven instructions represented actual measures being communicated to the German public at the time of data collection (e.g., “distance: Keep a distance of at least 1.5 meters to other people”), while two reflected public discourse regarding future measures (“compulsory vaccination” and “quarantine”). The instructions addressed aspects of daily life (e.g., “hygiene”) and specific situations (e.g., “holiday restriction”) and covered different levels of restriction. The English counterparts of the German instructions were created by a process of translation and back-translation.

**Table 1:** COVID-19 mitigation instructions used as stimuli.

	English (foreign language)	German (native language)
Distance	Keep a distance of at least 1.5 meters to other people.	Halten Sie mindestens 1,5 Meter Abstand zu anderen Personen.
MNC in public	Wear a mouth-nose-cover on public transport and when shopping.	Tragen Sie eine Mund-Nasen-Bedeckung in öffentlichen Verkehrsmitteln und beim Einkaufen.
Hygiene	Follow the rules of hygiene when sneezing, coughing and washing your hands.	Befolgen Sie die Hygieneregeln beim Niesen, Husten und Händewaschen.
MNC in restaurants	Wear a mouth-nose-cover when entering and leaving restaurants and bars.	Tragen Sie eine Mund-Nasen-Bedeckung beim Betreten und Verlassen von Restaurants und Bars.
COVID-19-app	Install the free-of-charge corona-app of the German government on your smartphone.	Installieren Sie die kostenfreie Corona-App der Bundesregierung auf Ihrem Smartphone.
Restriction of leisure activities	Avoid leisure activities in groups over ten people.	Vermeiden Sie Freizeitbeschäftigungen in Gruppen über zehn Personen.
Holiday restriction	Avoid holiday trips outside of Germany.	Vermeiden Sie Urlaubsreisen außerhalb Deutschlands.
Compulsory vaccination	To be able to participate fully in public life, you must get vaccinated against the coronavirus. Otherwise, you may be banned from attending events and places with large crowds.	Um uneingeschränkt am öffentlichen Leben teilnehmen zu können, müssen Sie sich gegen das Coronavirus impfen lassen. Andernfalls kann Ihnen der Besuch von Veranstaltungen und Orten mit großen Menschenansammlungen untersagt werden.
Quarantine	Since the number of coronavirus infections in your area is increasing rapidly, you will have to go into home quarantine for two weeks.	Da die Anzahl der Coronavirus Erkrankungen in ihrer Gegend schnell ansteigt, müssen Sie sich für zwei Wochen in häusliche Quarantäne begeben.

### Participants

Because human participants were involved in the study, additional measures were taken to ensure appropriate conduct. Upon clicking on the link to the online survey, respondents were informed that participation was voluntary and that they could stop the questionnaire at any time. They were informed that the survey was about getting their opinions on various pandemic mitigation instructions. Respondents were made aware that no personal data would be collected. By clicking the start button of the survey, participants consented to their participation. Our study was approved by the ethics committee of the university the authors are affiliated with. The study participants were recruited via an online microwork platform, and consistent with prior research on foreign language processing [e.g., 38], a student sample was used. This seemed appropriate because young people are likely to be active on social media, where much of the discourse on global events and crises is in English, which is a foreign language for many individuals [39]. In addition, a student sample represents a relatively homogenous group with similar life backgrounds and presumably largely similar attitudes and belief systems, which makes possible biases due to different perceptions of the pandemic less likely. This assumption is supported by the results of a large-scale study amongst German university students, which found that during the time frame of our study, a vast majority (85%) of students supported the governmental lockdown measures [40]. In addition, the respondents were native German speakers with a high proficiency in English to avoid confounding effects due to language comprehension. The participants were screened via self-evaluation of their English skills (7-point scale) and a text comprehension quiz [41]. Before being able to enter the main part of the survey, respondents who did not rate their English language proficiency with 5 or higher on the 7-point scale with the endpoints 7 = “very good knowledge” and 1 = “no knowledge at all” were screened out. This ensured that the messages were clear and understandable for each participant, regardless of the language condition. The questionnaire was completed by 631 respondents, of which 26 were removed because German was not their native language, they were not students, or they had performed poorly on the comprehension quiz. Therefore, the final sample consisted of 605 participants (*M*_*age*_ = 25.78, *SD* = 4.43; 47.3% female). On average, participants started learning the English language at the age of 9.36 and there was no significant difference regarding the age of acquisition between the respondents in the two research conditions (*M*_*NL*_ = 9.20 vs. *M*_*FL*_ = 9.57, *F*_*1*,*603*_ = 1.701, *p* = .193). Table 2 provides an overview of the participant demographics.

**Table 2:** Demographic details of study participants.

Characteristic	Survey respondents (*n* = 605)
Gender	
Female	47.27%
Male	52.07%
Diverse	0.66%
Age (Mean)	25.78 (*SD* = 4.43)
Highest education	
Secondary school diploma	44.46%
Bachelor’s degree	41.32%
Master’s degree	10.25%
Other	3.97%
Current occupation: student	100%
Nationality	
German	98.68%
German and other	.33%
Not German	.99%
Native language	
German	98.84%
German & other	1.16%

*Note*: Due to rounding, some columns do not add up to 100%.

### Procedure

The online survey took place between July and October 2020. During this period, various behavioral recommendations, for example, regarding hygiene rules or travel restrictions, were regularly issued by the German government. After being randomly assigned to one of the two language conditions, the participants provided demographic data and language skill information. Next, each participant evaluated the nine instructions in randomized order–either in English or German, depending on their assignment to the experimental condition. Finally, the participants provided additional information about their English proficiency. Following prior research, the entire questionnaire was presented either in German (native language) or in English (foreign language).

### Measures

**Fig 1:**
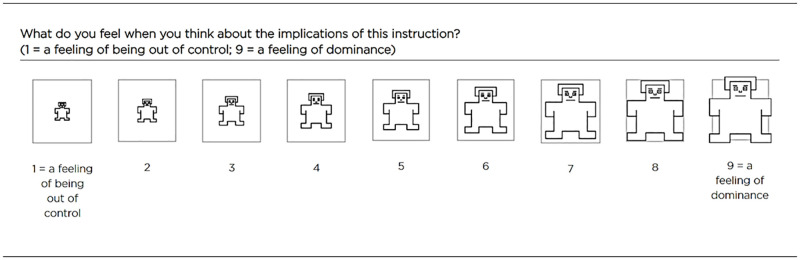
Sense of control item used in the study supplemented with a Self-Assessment Manikin [13].

To measure sense of control, we used a nine-point bipolar scale (“What do you feel when you think about the implications of this instruction?” 1 = “a feeling of being out of control,” 9 = “a feeling of dominance”) proposed by Lang [13]. Originally, this construct was named dominance. However, the terminology used in the items is that people are asked for their agreement as to how much in control they feel. We decided to use this scale because it was supplemented with images from the Self-Assessment Manikin [13], which has been used previously in research on foreign language processing [23]. The presence of emoticons is an important benefit of this scale because it mitigates a potential anchor contraction effect, which describes the tendency to report more intense emotions on rating scales in a foreign language that can bias the results [42]. The exact scale used in the questionnaire is depicted in Fig 1.

We assessed the participants’ cognitive evaluation of the effectiveness of the pandemic mitigation measures using two items proposed by Peck and Childers [43] (“How confident are you that this measure contributes to the containment of the coronavirus?”, 1 = “not very sure,” 9 = “very sure” and 1 = “not very confident,” 9 = “very confident”; *r* = .94). Behavioral intentions to adhere to the instruction were measured using two items rated on nine-point Likert scales (“It is very likely that I will follow this instruction” and “I think it makes sense to follow this instruction”; 1 = “strongly disagree,” 9 = “strongly agree”; *r* = .88). All measures were assessed after each instruction individually.

In addition, demographics and some control variables were assessed (e.g., gender, age, native language, nationality, and self-evaluation of all language skills of the individual). Because some studies reported the attenuation of emotions to be a driver of differences in foreign language processing [26, 27], we included emotional intensity (“How emotional is this instruction?”, 1 = not emotional, 7 = emotional) and arousal (emoticon supplemented scale by Lang [13]; 1 = “very calm,” 7 = “very aroused”) as additional measures.

### Statistical analyses

All study data was analyzed using the software IBM SPSS Statistics 27. Initially, descriptive analyses were used to describe the sample characteristics. Then, two repeated measure ANOVAs were conducted to compare sense of control, arousal, and perceived emotional intensity between the two language conditions. The nine instructions were included as the within-subjects factor while language, i.e., native (NL) versus foreign (FL), served as the between-subjects factor. Subsequently, mediation analyses were conducted using Hayes’ PROCESS macro (Version 4.1) which employs a bootstrapping method to estimate a confidence interval [44]. The indirect effect through the mediator is established if the 95% bootstrap interval does not include 0. First, PROCESS Model 4 was used to calculate two single mediation models with language condition (0 = native language, 1 = foreign language) as the independent variable, sense of control as the mediator and cognitive evaluation of the instructions’ effectiveness and the behavioral intention as the respective dependent variables. As part of additional analyses, PROCESS Model 7 was used to explore the conditional indirect effects of the language condition through sense of control for each individual instruction. In addition to the previously mentioned variables, Model 7 includes a moderator for the relationship between the independent variable and the mediator. Here, a variable classifying the nine individual instructions was used to estimate nine conditional indirect effects. All mediation analyses were run using 5,000 bootstrap samples.

## Results

### Direct effect of language

Our first prediction was that processing pandemic mitigation instructions in a foreign language would cause higher levels of sense of control. To test this assumption, we compared the two language conditions—native language (NL) versus foreign language (FL)—across the nine pandemic mitigation instructions. A one-way repeated measure ANOVA with language as a between-subject factor and nine measures per person for the dependent variable (one for each of the instructions) revealed a significantly higher sense of control (*M*_*NL*_ = 4.46 vs. *M*_*FL*_ = 4.72, *F*_*1*,*603*_ = 7.334, *p* = .007, partial η^2^ = .012) in the foreign language condition. The effect size was consistent with previous studies on foreign language processing [e.g., 17, 45].

This effect does not seem to be driven by an attenuation of emotions. We additionally compared the arousal elicited by the instructions and their perceived emotional intensity across the native and foreign language conditions using the same type of ANOVAs as described above. The results revealed no differences, neither in terms of arousal (*M*_*NL*_ = 4.06 vs. *M*_*FL*_ = 3.94, F_1,603_ = 1.183, *p* = .277, partial *η*^*2*^ = .002) nor in terms of emotional intensity (*M*_*NL*_ = 4.60 vs. *M*_*FL*_ = 4.70, F_1,603_ = .825, *p* = .364, partial *η*^*2*^ = .001).

### Mediation of sense of control on cognitive evaluation and behavioral intention

Moreover, sense of control was expected to mediate the effect of foreign language processing on the cognitive evaluation of the instructions’ effectiveness and the behavioral intention to comply with them. To test this assumption, we used bootstrapping to run two mediation models (Model 4: 5,000 samples, 95% CI, [44]; 0 = native language, 1 = foreign language). To test the model across all instructions, the values for sense of control, cognitive evaluation and behavioral intention were averaged. Consistent with our expectations, sense of control mediated the relationship between language and cognitive evaluation (*b* = .13, 95% CI [.03, .23]) of the instructions.

**Fig 2:**
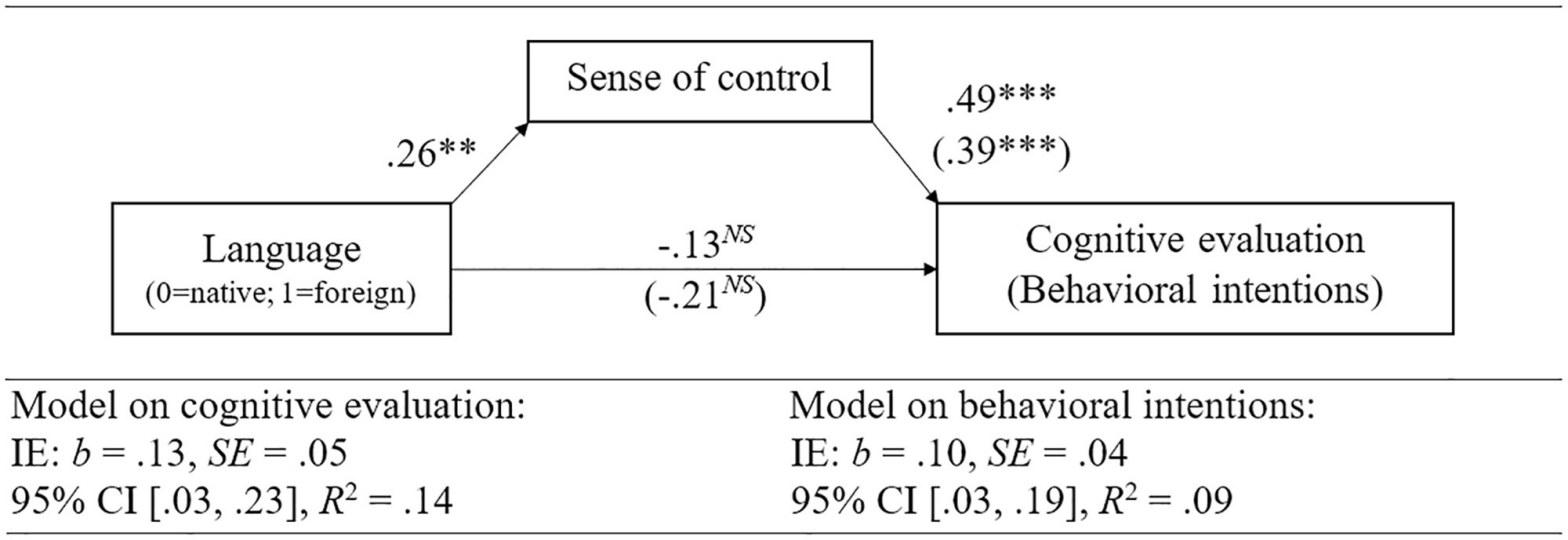
Mediating effects of sense of control on the cognitive evaluation of the pandemic mitigation measures and behavioral intentions (all instructions). Notes: IE = indirect effect, *b =* unstandardized regression coefficient. Results without brackets = model on cognitive evaluation, results in brackets = model on behavioral intentions. **p = .007, ***p < .001. *N* = 605. Model 4, 5,000 samples, 95% CI, [44].

Following the same rationale as for cognitive evaluation, we posited that sense of control will mediate the effect of foreign language processing on behavioral intentions. In line with our assumptions, sense of control mediated the relationship between language and behavioral intentions (*b* = .10, 95% CI [.03, .19]). While the predicted mediations through sense of control could be observed, there was no significant main effect of foreign language processing on cognitive evaluation and behavioral intentions. Both mediation models are depicted in Fig 2.

### Additional analysis

To learn which instructions mainly drive the observed effect, we calculated an additional mediation model in which the nine instructions were included as a moderating factor for the effect of language on sense of control (Model 7: 5,000 samples, 95% CI, [44]; 0 = native language, 1 = foreign language). The use of a moderated mediation model allowed us to determine for which of the individual instructions the observed mediation occurs. The results showed that foreign language processing led to an increased sense of control for the instructions “restriction of leisure activities”, “holiday restriction”, “compulsory vaccination”, and “quarantine”, and also led to the significant effect across all aggregated instructions. The detailed results for these instructions can be inspected in Table 3. No significant results were found for the remaining instructions covering “distance,” “mouth-nose cover in public,” “hygiene,” “mouth-nose cover in restaurants,” and “COVID-19-app.”

**Table 3:** Detailed bootstrapping results for the instructions that show the expected effects.

	Conditional effect on sense of control	Conditional IE on cognitive evaluation	Conditional IE on behavioral intentions
Restriction of leisure activities	*b* = .41, p = .0103 < .05	*b* = .13	*b* = .16
	95% CI [.10, .72]	95% CI [.04, .22]	95% CI [.05, .28]
Holiday restriction	*b* = .38, p = .0174 < .05	*b* = .12	*b* = .15
	95% CI [.07, .69]	95% CI [.02, .21]	95% CI [.03, .26]
Compulsory vaccination	*b* = .46, p = .0039 < .01	*b* = .14	*b* = .18
	95% CI [.15, .77]	95% CI [.03, .26]	95% CI [.04, .33]
Quarantine	*b* = .61, p < .001	*b* = .19	*b* = .24
	95% CI [.30, .92]	95% CI [.08, .30]	95% CI [.11, .37]

Notes: IE = indirect effect, *b* = unstandardized regression coefficient. Model 7, 5,000 samples, 95% CI, [44]. The instructions “distance”, “MNC in public”, “hygiene”, “MNC in restaurants”, and “COVID-19-app” were not included as the respective results were not significant.

The same four instructions–“restriction of leisure activities”, “holiday restriction”, “compulsory vaccination”, and “quarantine”–showed significant conditional indirect effects on both cognitive evaluation and behavioral intention. No significant mediations were found for “distance,” “mouth-nose cover in public,” “hygiene,” “mouth-nose cover in restaurants,” and “COVID-19-app.” Additional analyses showed that the four instructions for which significant mean-value differences and mediations were found were associated with higher levels of emotional intensity than the remaining five instructions (*M* = 3.78 vs. *M* = 5.71, *t*_*1*,*604*_ = -29.81, *p* < .001).

## Discussion

### Foreign language response to pandemic mitigation instructions

Our results suggest a higher sense of control when confronted with pandemic mitigation instructions in a foreign language as opposed to the recipient’s native language. Furthermore, we found that the effect of the foreign language on the recipient’s sense of control positively mediated not only behavioral intentions but also shifted the cognitive assessment of a given situation toward a higher confidence in the effectiveness of the instructions. As a result of higher sense of control, compliance with instructions that are freedom-restrictive to a comparatively high degree is considered more useful and purposeful by the recipients.

### Theoretical contributions

First, we contribute to the literature on crisis communication by showing that the mere usage of a different language changed the response to instructions that were to be obeyed, even though the content was translated to convey the same meaning in both languages. We observed a change in perceived control, which led to an improved belief in the effectiveness of freedom-threatening public health instructions and a higher intention of complying with them. This is a new finding in the health and crisis communication literature that had not been previously documented. It is consistent, though, with the proposed outcomes in Schroeder and Chen’s [46] literature analysis. These authors suggest that using a foreign language decreases fear and anxiety when processing health- and disease-related information, as well as increases rational decision-making.

Moreover, our results show that the described effects were mainly observed for instructions communicating more extreme restrictions or actions (e.g., “compulsory vaccination” and “quarantine”), whereas less extreme measures or regulations (e.g., “distance” and “mouth-nose cover”) are not affected by the difference in processing due to the foreign language. These results imply that the effect only shows for instructions that pose a greater threat to personal freedom: a compulsory vaccination or living in quarantine is far more intrusive into one’s personal life than wearing a mouth-nose cover in public or keeping distance from other people. It should be taken into account that the study was conducted at an early stage of the pandemic when compulsory vaccinations seemed very unlikely and extreme for a majority of people. This seems, however, consistent with foreign language research in other real-world contexts, where differences have been observed in studies with similarly “extreme” stimuli, such as vaccinations [19], insect-based food [25], crimes [18], or medical conditions [17].

On a more general level, it seems that using a foreign language makes people feel more capable when faced with freedom-restricting and potentially threatening messages and situations. People seem to show a different evaluation of their own selves, associated with higher levels of confidence. This finding appears to be related to recent foreign language research in the COVID-19 context. Geipel et al. [19] showed that hesitancy in being vaccinated against the coronavirus can be reduced by using a foreign language through increased trust in the vaccine. Our new finding of an enhanced sense of control in a foreign language seems to complement this. Both trust and a sense of control could be considered signals of security for individuals confronted with uncertain circumstances [e.g., 10, 47].

Furthermore, we offer several contributions to the literature on foreign language use. Previous foreign language research has largely used hypothetical or abstract scenarios [e.g., 21, 23]. In a currently relevant, real-life context that mirrors a freedom-restricting situation, we find that foreign language processing leads to differences in the perceived sense of control that carries over to people’s cognitive and conative responses. More specifically, higher levels of control translate into more positive cognitive evaluations of the pandemic mitigation instructions and a higher intention to comply with them. Furthermore, we add to the literature by demonstrating the influence of foreign language in an instructional context, in other words, for messages that directly address people to act in a certain way.

Moreover, we identified sense of control as a mediator between foreign language and both cognitive evaluation and behavioral intention. In the literature to date, there are differing interpretations of the consequences of foreign language processing, with some papers describing an attenuation of the emotional reaction [e.g., 22, 29] and others describing a shift toward a more positive reaction [e.g., 18, 25, 28]. Our results support the latter understanding by additionally showing that the experienced arousal—that is, the activating potential of the affective response—and the emotional intensity of the instructions were not significantly diminished in a foreign language, but there was still a difference in perceived control. Based on these results, we can cautiously suggest that the difference in perceived control does not merely stem from an attenuation of emotions but involves changes in the evaluation that include cognitive aspects. However, although not driving the effect, affective components seem to play a contextual role, because the effect occurs for those four instructions that are higher in their emotional intensity, such as “compulsory vaccination” and “quarantine” (emotional intensity: *M* = 5.71, *SD* = 1.63), compared to the five instructions lower in emotional intensity (*M* = 3.78, *SD* = 1.58).

Another point to be mentioned here is that sense of control may involve cognitive aspects [e.g., 48] as well as affective components [e.g., 49] that may both have been affected. More generally, to substantiate the assumption that the changes in evaluative judgments encompass both cognitive and affective components, we may refer to the organization of human memory. Schema and categorization theories assume that in the mental representation of semantic and episodic knowledge, affective tags are attached, representing positive or negative associations derived from prior experiences [e.g., 50, 51]. Possibly, in a foreign language, the way of how people cognitively access and use this knowledge stored in memory, including its affective tags, might be different (for example, less automatic). This may explain why changes in evaluations might involve affective components, although we did not measure differences in the immediate, situational experience of affect in our experiment. In addition, research on mental imagery may also contribute to this discussion. Mental imagery has been shown to be less vivid in a foreign language compared to the native language and seems to be dependent on both access to emotions via episodic memory [52, 53], but also cognitive capabilities such as recall, flexible recombination or reconstruction of memories [e.g., 54].

### Relevance and implications

Our findings show practical relevance in the context of global crises. They also seem highly relevant against the background of recent findings showing that a sense of control can serve as a protective mechanism for mental health during a global crisis [55]. A high sense of control has been linked to a high perceived ability to avoid uncertainty in a pandemic [56]. With our study, we expand upon the findings from Geipel et al. [19]. The use of a foreign language not only influences the decision to become vaccinated against the coronavirus [19]; it can also change the way individuals react to other publicly communicated pandemic mitigation measures.

From a managerial point of view, this study is highly relevant for all entities responsible for communicating (uncomfortable) appeals to the public. This may include, but is not limited to, policy makers, public institutions and offices, global employers, brands, and news agents. Our findings may help all entities that communicate freedom-threatening instructions better understand and predict the reactions of their recipients. Against the background of increasing amounts of content consumed in a foreign language, for example, in social media, this is of high importance. With global issues such as pandemics, environmental and economic crises, and increasing migration and globalization, numerous situations in which people are confronted with such freedom-threatening messages in a foreign language may increase. Importantly, our sample shows the effectiveness of foreign language communication in a target group that is very likely to be confronted with foreign language content on a regular basis: young people with excellent English skills. Generations Y and Z are increasingly communicating via global English-dominated platforms, such as TikTok and Instagram, especially in times of social distancing [57].

Finally, it should be noted that the effects elicited by the use of a foreign language presuppose that the content conveyed is clearly understood. This implies that not all health-related communication to all audiences should be delivered in a foreign language because it cannot be guaranteed that people have broad knowledge of a foreign language. Nevertheless, our study does serve as a recommendation for sporadic and targeted foreign-language communication in public health matters.

### Limitations and future research directions

Finally, this study is not without limitations. We measured behavioral intentions to assess whether recipients are more or less inclined to follow pandemic mitigation instructions in a foreign language. However, these self-reported measures cannot fully predict actual behavior. Thus, it remains to be verified whether changes in the perceived sense of control caused by foreign language processing lead to more compliant behavior. Furthermore, our results are based on a single sample of native German speakers. We used English, presumably representing one of the most practically relevant foreign languages in the context of current crisis communication. However, future studies should examine whether the effects occur regardless of the recipients’ native language (beyond the German in the current study). In addition, a possible bias due to prior opinions about the pandemic cannot be fully ruled out, although we reduced the risk to a minimum by using a large and homogenous sample of German students, which have been shown to hold a similar stance towards COVID-19 related governmental restrictions [40]. As previously noted, even though there was a positive mediation effect through sense of control, foreign language processing did not cause a significant main effect on cognitive evaluation and behavioral intentions. This suggests that parallel mediations related to native vs. foreign language processing additionally influence individual’s reactions toward instructions. It can be speculated that the respondents’ familiarity with the concrete instructions may play a role. The participants likely were more familiar with the native language instructions as they had already been extensively discussed in the media and in public discourse, potentially leading to a more favorable stance toward them. This may be one possible explanation why participants in the native language condition did not report a poorer cognitive evaluation and lower behavioral intentions, despite having a lower sense of control. Future research could address this issue.

Our results provide suggestions for future research directions. They suggest that people do not feel more secure in their native language when confronted with pandemic mitigation instructions. Our finding of a heightened sense of control with regard to a specific situation might point to a foreign language’s potential to increase people’s general perceived self-efficacy [58]. Another aspect is that the overall less threatening perception of messages in a foreign language could be due to the fact that, although proficiency in the foreign language is high, not as many layers of meaning of foreign words or phrases may unfold compared to native language processing (i.e., not as many semantic associations are drawn compared to native language processing). The richness or quantity of associations may not be completely accessed, as was previously suggested in the literature [17, 59]. People could have more access to the actual scope of restriction by instructions in their native language. Therefore, future research should investigate the relationship between the number of activated semantic associations in a native language and a foreign language.

## References

[1] Carothers T, Press B. The Global Rise of Anti-Lockdown Protests—And What to Do about It. World Politics Review. 2020 January 10 [Cited 2022 January 10]. Available from: https://www.worldpoliticsreview.com/articles/29137/amid-the-covid-19-pandemic-protest-movements-challengelockdowns-worldwide.

[2] Wiest B. The Psychological Reason Why Some People Aren’t Following COVID-19 Quarantine Orders. Forbes. 2020 April 8 [Cited 2022 February 15]. Available from: https://www.forbes.com/sites/briannawiest/2020/04/08/the-psychological-reason-why-some-people-arent-following-covid-19-quarantine-orders/?sh=45cd4fe86905.

[3] Gray L, MacDonald C, Mackie B, Paton D, Johnston D, Baker MG. Community responses to communication campaigns for influenza A (H1N1): a focus group study. BMC Public Health. 2012;12(1): 1–12. doi: 10.1186/1471-2458-12-205 22429559 PMC3324376

[4] Shoenberger H, Kim E, Sun Y. Advertising during COVID-19: exploring perceived brand message authenticity and potential psychological reactance. J Advert. 2021;50(3): 253–261.

[5] Heffner J, Vives ML, Feldman-Hall O. Emotional responses to prosocial messages increase willingness to self-isolate during the COVID-19 pandemic. Pers Individ Differ. 2021;170: 110420. doi: 10.1016/j.paid.2020.110420 33082614 PMC7561320

[6] Franceschini C, Musetti A, Zenesini C, Palagini L, Scarpelli S, Quattropani MC, et al. Poor Sleep Quality and Its Consequences on Mental Health During the COVID-19 Lockdown in Italy. Front Psychol. 2020;11: 574475. doi: 10.3389/fpsyg.2020.574475 33304294 PMC7693628

[7] Killgore WDS, Cloonan SA, Taylor EC, Allbright MC, Dailey NS. Trends in suicidal ideation over the first three months of COVID-19 lockdowns. Psychiatry Res. 2020;293: 113390. doi: 10.1016/j.psychres.2020.113390 32835926 PMC7430225

[8] Arendt F, Markiewitz A, Mestas M, Scherr S. Covid-19 pandemic, government responses and public mental health: Investigating consequences through crisis hotline calls in two countries. Soc Sci Med. 2020;265: 113532. doi: 10.1016/j.socscimed.2020.113532 33223385

[9] Wnuk A, Oleksy T, Maison D. The acceptance of Covid-19 tracking technologies: The role of perceived threat, lack of control, and ideological beliefs. PLOS One. 2020;15(9): e0238973. doi: 10.1371/journal.pone.0238973 32915908 PMC7485859

[10] Zhu N, O J, Lu HJ, Chang L. Debate: Facing uncertainty with (out) a sense of control–cultural influence on adolescents’ response to the COVID‐19 pandemic. Child Adolesc Ment Health. 2020;25(3): 173–174. doi: 10.1111/camh.12408 32681578 PMC7405212

[11] Mühlberger C, Jonas E, Sittenthaler S. Uncontrollability, reactance, and power: Power as a resource to regain control after freedom threats. In: Bukowski M, Fritsche I, Guinote A, Kofta M, editors. Coping With Lack of Control In A Social World. London: Routledge, 2016: pp. 230–246.

[12] Kay AC, Whitson JA, Gaucher D, Galinsky AD. Compensatory control: Achieving order through the mind, our institutions, and the heavens. Curr Dir Psychol Sci. 2009;18(5): 264–268.

[13] Lang P. Behavioral treatment and bio-behavioral assessment: computer applications. In: Johnson JH, Williams TA, Sidowski JB, editors. Technology in mental health care delivery systems. Norwood, NJ: Ablex; 1980: 119–37.

[14] Russell JA, Mehrabian A. Evidence for a three-factor theory of emotions. J Res Pers. 1977;11(3): 273–294.

[15] Bandura A. Self-efficacy: Toward a unifying theory of behavioral change. Psychol Rev. 1977;84(2): 191–215. doi: 10.1037//0033-295x.84.2.191 847061

[16] Fiske ST, Taylor SE. Social cognition. Mcgraw-Hill Book Company; 1991.

[17] Hayakawa S, Pan Y, Marian V. Language changes medical judgments and beliefs. Int J Biling. 2021;26(1): 104–121. doi: 10.1177/13670069211022851 35509268 PMC9060288

[18] Woumans E, Van der Cruyssen I, Duyck W. Crime and punishment: Morality judgment in a foreign language. J Exp Psychol Gen. 2020;149(8): 1597–1602. doi: 10.1037/xge0000736 31928022

[19] Geipel J, Grant LH, Keysar B. Use of a language intervention to reduce vaccine hesitancy. Sci Rep. 2022;12: 253. doi: 10.1038/s41598-021-04249-w 34997145 PMC8742025

[20] Geipel J, Hadjichristidis C, Surian L. The foreign language effect on moral judgment: the role of emotions and norms. PLOS One. 2015b;10(7): e0131529. 26177508 10.1371/journal.pone.0131529 PMC4503530

[21] Dylman AS, Bjärtå A. When your heart is in your mouth: the effect of second language use on negative emotions. Cogn Emot. 2019;33(6): 1284–1290. doi: 10.1080/02699931.2018.1540403 30384794

[22] Keysar B, Hayakawa SL, An SG. The foreign-language effect: thinking in a foreign tongue reduces decision biases. Psychol Sci. 2012;23(6): 661–668. doi: 10.1177/0956797611432178 22517192

[23] Hadjichristidis C, Geipel J, Surian L. Breaking magic: foreign language suppresses superstition. Q J Ex Psychol. 2019;72(1): 18–28. doi: 10.1080/17470218.2017.1371780 28835157

[24] Champoux-Larsson MF, Knežević Cvelbar L. Pro-environment choices using a second language. Ann Tour Res. 2020;89: 103089.

[25] Geipel J, Hadjichristidis C, Klesse AK. Barriers to sustainable consumption attenuated by foreign language use. Nat Sustain. 2018;1(1): 31–33.

[26] Dewaele JM. The emotional force of swearwords and taboo words in the speech of multilinguals. J Multiling Multicult Dev. 2004;25(2–3): 204–222.

[27] Puntoni S, De Langhe B, Van Osselaer SM. Bilingualism and the emotional intensity of advertising language. J Consum Res. 2009;35(6): 1012–1025.

[28] Hadjichristidis C, Geipel J, Savadori L. The effect of foreign language in judgments of risk and benefit: the role of affect. J Exp Psychol Appl. 2015;21(2): 117–129. doi: 10.1037/xap0000044 25893443

[29] Costa A, Foucart A, Arnon I, Aparici M, Apesteguia J. Piensa” twice: on the foreign language effect in decision making. Cogn. 2014;130(2): 236–354. doi: 10.1016/j.cognition.2013.11.010 24334107

[30] Białek M, Muda R, Stewart K, Niszczota P, Pieńkosz D. Thinking in a foreign language distorts allocation of cognitive effort: Evidence from reasoning. Cogn. 2020;205: 104420. 10.1016/j.cognition.2020.104420 33032818

[31] Mækelæ M, Pfuhl G. Deliberate reasoning is not affected by language. PLOS One. 2019;14: e0211428. doi: 10.1371/journal.pone.0211428 30703137 PMC6355010

[32] Geipel J, Hadjichristidis C, Surian L. Foreign language affects the contribution of intentions and outcomes to moral judgment. Cogn. 2016;154: 34–39. doi: 10.1016/j.cognition.2016.05.010 27232522

[33] Ajzen I. Perceived behavioral control, self-efficacy, locus of control, and the theory of planned behavior. J Appl Soc Psychol. 2002;32(4): 665–683.

[34] Leyro TM, Zvolensky MJ, Bernstein A. Distress tolerance and psychopathological symptoms and disorders: A review of the empirical literature among adults. Psychol Bull. 2010;136(4): 576–600. doi: 10.1037/a0019712 20565169 PMC2891552

[35] Xu Q, Kwan CM, Zhou X. Helping yourself before helping others: How sense of control promotes charitable behaviors. J Consum Psychol. 2020; 30(3): 486–505.

[36] Folkman S. Personal control and stress and coping processes: A theoretical analysis. J Pers Soc Psychol. 1984;46(4): 839–852. doi: 10.1037//0022-3514.46.4.839 6737195

[37] Rogers RW. Cognitive and physiological processes in fear appeals and attitude change: A revised theory of protection motivation. In: Petty RE, Cacioppo JT, editors. Social psychophysiology. New York: Guilford Press; 1983. pp. 153–176.

[38] Geipel J, Hadjichristidis C, Surian L. How foreign language shapes moral judgment. J Exp Soc Psychol. 2015a, 59: 8–17.

[39] Clark E, Araki K. Text normalization in social media: progress, problems and applications for a pre-processing system of casual English. Procedia Soc. 2011;27: 2–11.

[40] Kohls E, Baldofski S, Moeller R, Klemm SL, Rummel-Kluge C. Mental health, social and emotional well-being, and perceived burdens of university students during COVID-19 pandemic lockdown in Germany. Front Psychiatry. 2021;12: 643957. doi: 10.3389/fpsyt.2021.643957 33889102 PMC8055863

[41] Hayakawa S, Tannenbaum D, Costa A, Corey JD, Keysar B. Thinking more or feeling less? Explaining the foreign-language effect on moral judgment. Psychol Sci. 2017;28(10): 1387–1397. doi: 10.1177/0956797617720944 28806137

[42] De Langhe B, Puntoni S, Fernandes D, Van Osselaer SM. The anchor contraction effect in international marketing research. J Mark Res. 2011;48(2): 366–380.

[43] Peck J, Childers TL. To have and to hold: the influence of haptic information on product judgments. J Mark. 2003;67(2): 35–48.

[44] Hayes AF. Introduction to mediation, moderation, and conditional process analysis: A regression-based approach. New York: Guilford Press; 2018.

[45] Circi R, Gatti D, Russo V, Vecchi T. The foreign language effect on decision-making: a meta-analysis. Psychon Bull Rev. 2021;28(4): 1131–1141. doi: 10.3758/s13423-020-01871-z 33555512

[46] Schroeder SR, Chen P. Bilingualism and COVID-19: using a second language during a health crisis. J Commun Healthc. 2021;14(1): 20–30.

[47] Spadaro G, Gangl K, Van Prooijen J-W, Van Lange PAM, Mosso CO. Enhancing feelings of security: How institutional trust promotes interpersonal trust. PLOS One. 2020;15(9): e0237934. doi: 10.1371/journal.pone.0237934 32916694 PMC7486136

[48] Keeton CP, Perry-Jenkins M, Sayer AG. Sense of control predicts depressive and anxious symptoms across the transition to parenthood. J Fam Psychol. 2008;22(2):212–221. doi: 10.1037/0893-3200.22.2.212 18410208 PMC2834184

[49] Perloff LS. Perceptions of vulnerability to victimization. J Soc Issues. 1983;39(2): 41–61.

[50] Fiske ST, Pavelchak MA. Category-based versus piecemeal-based affective responses: Developments in schema-triggered affect. In: Sorrentino RM, Higgins ET, editors. Handbook of motivation and cognition: Foundations of social behavior. New York: Guilford Press, 1986: pp. 167–203.

[51] Fiske ST, Neuberg SL, Beattie AE, Milberg SJ. Category-based and attribute-based reactions to others: Some informational conditions of stereotyping and individuating processes. J Exp Soc Psychol. 1987;23(5): 399–427.

[52] Hayakawa S, Keysar B. Using a foreign language reduces mental imagery. Cogn. 2018;173: 8–15. doi: 10.1016/j.cognition.2017.12.010 29278805

[53] Tulving E. Memory and consciousness. Can Psychol. 1985;26(1): 1–12.

[54] Schacter D, Addis D, Buckner RR. Remembering the past to imagine the future: the prospective brain. Nat Rev Neurosci. 2007;8(9): 657–661. doi: 10.1038/nrn2213 17700624

[55] Xiong P, Ming W, Zhang C, Bai J, Luo C, Cao W, et al. Factors influencing mental health among Chinese medical and non-medical students in the early stage of COVID-19 pandemic. Front Public Health. 2021;9: 603331. 34095044 10.3389/fpubh.2021.603331 PMC8172592

[56] Peluso AM, Pichierri M. Effects of socio-demographics, sense of control, and uncertainty avoidability on post-COVID-19 vacation intention. Curr Issues Tour. 2021;24(19): 2755–2767.

[57] Bowden-Green T, Hinds J, Joinson A. Personality and motives for social media use when physically distanced: a uses and gratifications approach. Front Psychol. 2021;12: 607948. doi: 10.3389/fpsyg.2021.607948 34194354 PMC8238001

[58] Schwarzer R, Warner LM. Perceived self-efficacy and its relationship to resilience. In: Saklofske DH, Prince-Embury S, editors. Resilience in children, adolescents, and adults. New York, NY: Springer; 2013. pp. 139–150.

[59] Caldwell-Harris CL. Emotionality differences between a native and foreign language: theoretical implications. Front Psychol. 2014;5: 1055. doi: 10.3389/fpsyg.2014.01055 25295019 PMC4172089

